# Exploring the Role of Professional Burnout and Mental Health Strain in Nurses’ Turnover Intentions: A Mediation Study

**DOI:** 10.1155/jonm/1175290

**Published:** 2025-12-16

**Authors:** Ibrahim Abdullatif Ibrahim, Atallah Alenezi, Mennat Allah G. Abou Zeid, Mahmoud Abdelwahab Khedr, Ayman Mohamed El-Ashry, Bander Saad Albagawi, Samah Mohamed Abdelrahim

**Affiliations:** ^1^ Department of Nursing Sciences, College of Applied Medical Sciences, Shaqra University, Shaqra, Saudi Arabia, su.edu.sa; ^2^ Psychiatric and Mental Health Nursing Department, College of Nursing, Imam Mohammad Ibn Saud Islamic University (IMSIU), Riyadh, Saudi Arabia, imamu.edu.sa; ^3^ Nursing Administration and Education Department, College of Nursing, Prince Sattam Bin Abdulaziz University, Al-Kharj, Saudi Arabia, psau.edu.sa; ^4^ Psychiatric and Mental Health Nursing Department, Faculty of Nursing, Alexandria University, Alexandria, Egypt, alexu.edu.eg; ^5^ Medical Surgical Department, College of Nursing, Imam Mohammad Ibn Saud Islamic University (IMSIU), Riyadh, Saudi Arabia, imamu.edu.sa; ^6^ Department of Nursing Administration, Faculty of Nursing, Damietta University, Damietta, Egypt, du.edu.eg

**Keywords:** Egypt, job satisfaction, mediation analysis, mental health, nurses, personnel turnover, professional burnout, psychological stress

## Abstract

**Background:**

Nurses’ turnover intentions had a significant challenge to healthcare systems worldwide. Burnout substantially contributes to turnover intentions and actual departure behavior, yet the underlying mechanisms remain underexplored.

**Aim:**

To examine the mediating role of mental health strain in the relationship between professional burnout and nurses’ turnover intentions.

**Methods:**

This cross‐sectional study employed a nonprobability, convenience sample of 234 registered nurses working at two tertiary hospitals in the Damietta Governorate, Egypt. Out of the 255 questionnaires distributed, 234 were returned, yielding a 91.8% response rate. Data were collected using self‐reported questionnaires, including the Copenhagen Burnout Inventory (specifically work‐related burnout subscale), the General Health Questionnaire, and the Turnover Intentions Scale, in addition to a demographic information form. Data were analyzed using descriptive and inferential statistics followed by Hayes’ PROCESS Macro (Model 4) to conduct mediation analysis.

**Results:**

Professional burnout had a substantial positive direct influence on mental health strain (*B* = 0.221, *p* < 0.001) and turnover intentions (*B* = 0.589, *p* < 0.001). Mental health strain also substantially affected turnover intentions (*B* = 0.749, *p* < 0.001). Mediation analysis showed that mental health strain partially mediated the association between professional burnout and turnover intentions (*B* = 0.166; 95% CI [0.090–0.256]). Moreover, age (*B* = 0.067, *p* < 0.05) and experience (*B* = −0.078, *p* < 0.05) had a statistically significant influence on turnover intentions.

**Conclusions:**

The findings highlight mental health strain as a crucial pathway through which professional burnout affects turnover intentions among nurses. Nursing managers should implement strategies to reduce professional burnout and support nurses’ mental well‐being to enhance retention and workforce stability. Additionally, health policymakers should consider implementing national standards for safe staffing, institutionalized mental health support programs, and long‐term investments in workplace wellness to ensure a resilient and sustainable nursing workforce.

## 1. Introduction

The global healthcare workforce is facing an exceptional challenge. According to the World Health Organization (WHO), it is forecasted that by 2030, there will be a need for nine million nurses and midwives globally to attain the objective of health and well‐being [1]. As the backbone of healthcare systems, nurses play a critical role in direct patient care, clinical decision‐making, and upholding safety standards, often serving in situations where physicians are not immediately available [2]. Additionally, nurses must provide patients with high‐quality care around‐the‐clock, 7 days a week, as part of their duty to maintain a safety culture. Due to their extensive caregiving roles, nurses often work prolonged shifts and manage high patient loads, placing them at increased risk of professional burnout [3]. Professional burnout has been empirically linked to adverse patient outcomes including medication errors, healthcare‐associated infections, increased mortality, and reduced quality of care [4, 5]. Moreover, one of the most alarming organizational consequences of burnout is turnover intention, which intensifies staffing shortages, disrupts continuity of care, and increases recruitment and training costs [6, 7].

In Egypt, the prevalence of burnout and turnover intentions among nurses is alarmingly high. Empirical national studies have reported that nurses experience moderate to high levels of burnout, with many expressing intentions to leave their profession [8–14]. These trends have significant implications for an already strained healthcare system facing workforce shortages and escalating patient demands. Despite the growing urgency of this issue, limited research in the Egyptian context has examined the psychological mechanisms linking burnout to turnover intentions, particularly the mediating role of mental health strain. By addressing this gap, the present study contributes localized, evidence‐based insights necessary for designing contextually appropriate interventions and informing both organizational practices and national workforce policies.

Therefore, our study aims to address a critical gap by exploring the mediating effect of mental health strain on the relationship between professional burnout and turnover intentions among nurses. This mediating pathway is conceptually informed by two complementary theoretical frameworks: the Job Demands‐Resources (JD‐R) model and the Conservation of Resources (COR) theory.

The JD‐R model [15] explains how chronic job demands and insufficient resources contribute to professional burnout, resulting in mental health strain. This strain represents the immediate psychological consequences of energy depletion, which may increase turnover intentions as a behavioral response. In contrast, COR theory [16] focuses on the motivational processes underlying resource loss. From this perspective, burnout signals a significant depletion of valued resources (e.g., emotional energy and efficacy). Mental health strain reflects the perceived threat of further resource loss, which may motivate turnover intentions as a strategy to preserve remaining resources and prevent total depletion.

By grounding mental health strain within these theoretical frameworks, the current study advances the literature in two meaningful ways. First, it offers a more nuanced understanding of how burnout leads to turnover intention, moving beyond direct effects to explore mediating psychological processes. Second, it provides empirical insights that are highly relevant for nursing administrators, policymakers, and mental health advocates. Identifying mental health strain as a critical pathway from burnout to turnover allows healthcare institutions to design more targeted interventions such as psychological support services, resilience training, and workload redesign that protect nurses’ well‐being and reduce attrition.

## 2. Literature Review and Hypothesis Development

### 2.1. Professional Burnout in Nursing

Professional burnout, defined as “a condition of extended psychological and physical tiredness seen as connected to the person’s job” [17]. It is particularly prevalent in high‐stress healthcare settings such as nursing, where frontline caregivers are exposed to emotionally demanding situations, long shifts, heavy workloads, and limited autonomy [18]. Conceptually, burnout is typically understood through three key dimensions: emotional exhaustion, depersonalization, and reduced personal accomplishment [19]. Empirical evidence confirms that nurses experience higher levels of burnout than many other professions [20, 21]. Burnout among nurses is not merely an individual psychological outcome, but a complex, systemic response to sustained workplace pressures. These demands compromise both the physical and mental well‐being of nurses and adversely affect the quality and safety of patient care [18, 22]. In this context, burnout should be viewed not just as a personal issue, but as a broader organizational problem with serious consequences.

### 2.2. Professional Burnout and Turnover Intentions in Nursing

Turnover intention, conceptualized as “an employee’s desire to leave their current position within a specific time period” [23], is a critical outcome of burnout. Empirical evidence from South Korea further underscores a strong relationship between burnout, emotional exhaustion, and turnover intentions [24], suggesting that these constructs are interlinked. Notably, burnout does not operate in isolation. It often combines with other job stressors such as poor team dynamics, excessive workload, and lack of recognition, which cumulatively increase turnover intentions [25]. From a theoretical perspective, COR theory posits that individuals strive to acquire, retain, and protect resources they value (e.g., time, emotional energy, recognition) [16]. Burnout, in this theoretical frame, represents a state of resource depletion. As such, intention to leave may be a rational coping strategy, allowing nurses to protect what remains of their personal and professional resources. Thus, we propose the following:

 H1: Professional burnout will be positively associated with turnover intentions among nurses.

### 2.3. Professional Burnout and Mental Health Strain in Nursing

Mental health strain refers to psychological distress resulting from conflicting pressures or stressors [26], often manifesting as anxiety, depression, or emotional fatigue [27]. Unlike transient stress, mental health strain represents a more severe condition involving multiple concurrent stressors pulling an individual in opposing directions [28]. Research indicates that occupational burnout significantly contributes to this form of psychological distress. For example, Chen and Meier found that burnout was strongly linked to depressive symptoms among nurses [29]. Similarly, negative workplace environments were positively correlated with symptoms of depression, anxiety, and stress [30], reinforcing the idea that chronic occupational stress erodes mental well‐being. Importantly, the interaction between emotional exhaustion and the inability to cope with role demands exacerbates mental strain. Therefore, we hypothesize:

 H2: Professional burnout will be positively associated with mental health strain among nurses

### 2.4. Mental Health Strain and Turnover Intention in Nursing

The influence of mental health strain on employment decisions is profound, particularly in high‐stress professions like nursing. Psychological discomfort increases the likelihood of considering career withdrawal due to emotional fatigue and perceived inability to manage job demands [31, 32]. Anees et al. [33] reported a direct relationship between psychological strain and intent to quit, underscoring its role as a proximal determinant of withdrawal behavior. Moreover, Lapointe et al. [34] highlighted that strain reduces engagement and cultivates negative professional self‐concepts, key antecedents of turnover. From a theoretical perspective, the JD‐R model posits that when job demands exceed the employee’s coping resources, the resulting strain catalyzes disengagement and turnover [15]. Notably, Korbus et al. [35] highlighted how chronic exposure to job strain, such as understaffing and role ambiguity erodes psychological health and ultimately leads to turnover intentions. When mental reserves are continually depleted without adequate recovery, motivation decreases and turnover becomes a coping mechanism. Consequently, we formulated the following hypothesis:

 H3: Mental health strain will be positively associated with turnover intentions among nurses.

### 2.5. Mediating Role of Mental Health Strain

The JD‐R model suggests that persistent job demands first induce emotional exhaustion and strain, which then precipitate withdrawal behaviors [36]. This mechanism has received empirical support across various contexts. For instance, Obeng et al. [37] found that psychological strain fully mediated the relationship between job insecurity and turnover, highlighting how mental strain acts as a conduit through which job stressors lead to intent to quit. Giorgi et al. [38] further showed that strain mediates the relationship between workplace bullying and emotional regulation, pointing to its broader psychological implications. Furthermore, the COR theory [16] posits that individuals strive to obtain and maintain resources, and the loss of these resources leads to stress and burnout. In the nursing context, the depletion of emotional and psychological resources due to burnout can result in mental health strain, which in turn increases turnover intentions as nurses seek to safeguard their remaining resources. These findings collectively reinforce the view that mental health strain is not merely a consequence of burnout but serves as a critical mediating mechanism through which burnout influences adverse outcomes, such as nurses’ intentions to leave their profession. In light of these insights, the following hypothesis is proposed:

 H4: Mental health strain will mediate the relationship between professional burnout and turnover intentions.

**Figure 1:**
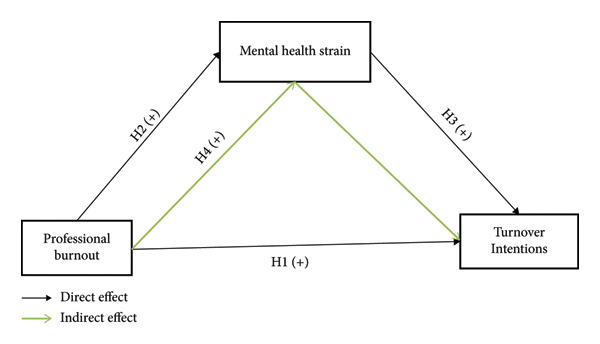
The conceptual model of the study.

Based on the aforementioned literature review and hypothesis development, the conceptual framework for this study is presented in Figure 1.

## 3. Methods

### 3.1. Study Design and Setting

This cross‐sectional research was carried out at two tertiary hospitals located in Damietta Governorate, Egypt. This hospital provides comprehensive healthcare services, including specialist units in surgery, internal medicine, critical care, and psychiatry, catering to varied patient demographics and offering therapeutic and diagnostic services. These hospitals were selected for their high patient load, diverse clinical departments, and representativeness of public healthcare settings in Egypt. Their complexity and workforce size made them suitable for examining burnout and turnover among nurses. The study adhered to STROBE reporting guidelines.

### 3.2. Data Collection Procedure

Data collection occurred over 2 months, from January to the end of February 2025. Questionnaires on paper were distributed to nurses during their working hours to enhance convenience and optimize participation rates. Before distributing the questionnaire, brief informational sessions were held to explain the ethical considerations, the study’s aim, and significance. Participants were assured that their responses would not affect their employment and professional standing. The participants were directed to examine their entire questionnaires to confirm that all questions were answered accurately and thoroughly, leading to a final dataset with no missing responses. Completed questionnaire were returned by participants in sealed envelopes to maintain confidentiality. These envelopes were collected and stored in locked containers locked containers in nursing managers’ offices.

### 3.3. Participants

The study population composed of nonprobability convenience sample of 234 registered nurses employed at the selected hospitals. Nurses from different units, levels of experience, and shifts were invited to participate, ensuring representation across various workplace conditions. Inclusion criteria required participants to have at least 1 year of clinical experience, work full‐time, and be directly involved in patient care. Exclusion criteria included nurses on leave, temporarily assigned to nonclinical duties during the data collection period, nursing interns, nurses with serious physical or mental illnesses, and retired nurses. The total number of eligible registered nurses at the two participating hospitals (*N* = 517) was obtained from the hospitals’ Human Resources offices. The sample size was calculated with G∗Power 3.1 software, employing a multiple regression model with an anticipated effect size of 0.15, a power of 0.80, and an alpha level of 0.05. A minimum sample of 153 nurses was required by these factors. To accommodate nonresponses and incomplete questionnaires, an extra 20% was included, resulting in a final target sample of 183 nurses. Of the 255 issued questionnaires, 234 nurses consented to participate, yielding a valid response rate of 91.8% for analysis.

### 3.4. Study Instruments

This study utilized three tools and a demographic questionnaire for data collection (Supporting File 1).

Professional burnout was evaluated using the 7‐item work‐related burnout subscale of the Copenhagen Burnout Inventory (CBI) [17]. This subscale focuses on exhaustion directly linked to workplace demands, workload, and professional responsibilities. Each item rated on a 5‐point Likert scale, with response options varying from “Never/a very low degree (1) to always/a very high degree (5).” Item 4 should be reverse‐scored; therefore, higher scores reflect greater levels of professional burnout. The full CBI includes additional subscales assessing personal and client‐related burnout. However, this study specifically targeted work‐related burnout directly addresses occupational stressors relevant to nursing practice. Utilizing this focused subscale reduces participants’ burden and enhances measurement precision regarding workplace‐related exhaustion which aligns with the study’s aim. The total score of the CBI work‐related burnout subscale ranged from 7 to 35.

Mental health strain was assessed using the General Health Questionnaire (GHQ) including 12‐item designed to evaluate mental health status [39]. Half of the items are positively phrased, while the other half is negatively phrased. Each item offers four possible response options. Scores for each item were coded using the Likert method (0‐1‐2‐3 scale). For the positively worded items, the scores were reverse coded to ensure that a greater score indicates a more adverse mental health condition, maintaining compatibility with the scale’s original meaning. The total score of the GHQ ranged from 0 to 36. Although GHQ originally developed as a screening tool for common mental disorders, GHQ has been extensively used in occupational health research as a measure of psychological distress, particularly among healthcare professionals [40, 41]. It captures core symptoms of distress, including anxiety, depressive mood, and social dysfunction, which align conceptually with the construct of “mental health strain” in the present study. Prior studies have demonstrated strong psychometric properties of the GHQ‐12, with Cronbach’s alpha values typically exceeding 0.85 in nursing [42]. Its brevity, sensitivity, and cross‐cultural applicability make it an appropriate and pragmatic tool for assessing mental health strain in high‐demand clinical contexts.

The three‐item turnover intentions scale adapted from Moosa et al. [43]. Responses from respondents were assessed using a 5‐point Likert scale, ranging from “strongly disagree” (1) to “strongly agree” (5). A higher score indicates a strong desire to leave. The total score of the turnover intentions ranged from 3 to 15.

The demographic characteristics questionnaire includes questions about the participants’ gender, age, education, marital status, and years of nursing experience.

### 3.5. Validity and Reliability

The study utilized standardized scales that were translated from English to Arabic and back‐translated using Brislin’s guidelines to accuracy and cultural relevance [44]. A panel of experts, comprising two professors of nursing management and one professor of mental health nursing, evaluated the content validity of the translated scales. A pilot study was then undertaken with 27 nurses to evaluate the clarity and application of the measures. The pilot study indicated no problems or concerns, affirming the clarity and suitability of the scales for the research. The sample from the pilot research was omitted from the final analysis.

The results of Cronbach’s alpha coefficient demonstrated high internal consistency, with the work‐related burnout scale yielding a Cronbach alpha of 0.88, while the GHQ yielded 0.90, and the turnover intentions scale yielded 0.86. All scales surpassed the specified level of 0.70, the acceptable grade for questionnaire reliability [45].

Confirmatory factor analysis (CFA) was conducted on the datasets using the statistical program AMOS 24 to evaluate construct validity. The model fit was assessed according to the criteria proposed by Hu and Bentler, which include chi‐square (*χ* ^2^), degrees of freedom (d*f*), *χ* ^2^/d*f* (< 5), comparative fit index (CFI > 0.90), Tucker–Lewis index (TLI > 0.90), root mean square error of approximation (RMSEA < 0.08), and standardized root mean square residual (SRMR < 0.08) [46]. The CFA for professional burnout exhibits robust fit indices: *χ* ^2^/DF = 2.18, CFI = 0.982, TLI = 0.968, RMSEA = 0.071, and SRMR = 0.042. Correspondingly, the CFA for mental health strain exhibits an exemplary fit, with *χ* ^2^/DF = 2.44, CFI = 0.931, TLI = 0.914, RMSEA = 0.07, and SRMR = 0.055. Finally, the CFA for turnover intentions shows an exceptional fit, with *χ* ^2^/DF = 1.46, CFI = 0.999, TLI = 0.996, RMSEA = 0.044, and SRMR = 0.014. The findings validate the construct validity of the measures and affirm their suitability for the research.

### 3.6. Ethical Considerations

Ethical approval for this study was granted by the Research Ethics Committee at Damietta University (Reference Number: DU Rec no 55; dated: 29/12/2024). The research complied with the ethical standards established in the Declaration of Helsinki. All participants provided written informed consent prior to data collection. It was explicitly stated in the form of consent that participation was voluntary and that they would not receive any compensation for their involvement. Participants’ identities were safeguarded through the use of coded identifiers for anonymization. Nurses were informed that they retained the right to withdraw at any point without penalty. Survey responses were stored in password‐protected files, accessible only to the research team.

### 3.7. Statistical Analysis

The normality assumption was assessed based on sample size exceeded 30, allowing reliance on the central limit theorem (CLT), which support normality for large samples [47]. Moreover, visual inspections using histograms showed no significant deviations from normality. Absolute *z*‐value for skewness and kurtosis values also fell with acceptable ranges further confirming normality [48]. Descriptive statistics summarized demographic characteristics and study variables, presenting results as means, standard deviations, frequencies, and percentages. The independent *t*‐test and ANOVA were employed to examine differences in demographic‐related variables within the study. Pearson’s correlation coefficients were employed to assess the relationships among professional burnout, mental health strain, and turnover intentions. The mediating effect of mental health strain (M) on the relationship between professional burnout (X) and turnover intentions (Y) was examined utilizing PROCESS macro version 4.2 beta (model 4). The statistically significant differences in the study variables–related demographics were controlled as covariates in mediation analysis. A biased bootstrap 95% confidence interval (CI) with 5000 samples to determine the significance of total, direct, and indirect effects did not include zero. The data was examined using IBM SPSS Statistics version 27 and AMOS 24.

## 4. Results

### 4.1. Participant Characteristics and Variations in Study Variables

The average age of the participants was 31.28 years, with a standard deviation of 8.04 years. A significant portion of the participants (59.4%) were between the ages of 20 and 30. The majority were female (88.9%) and married (67.1%). Most of the nurses in the study held a technical degree in nursing education (87.6%) and had between 1 and 5 years of work experience, which constituted 44.4% of the sample. The mean years of work experience among the participants was 10.11 years, with a standard deviation of 8.64 years.

Significant differences were found in professional burnout based on age group (*p* < 0.001), marital status (*p* < 0.05), and years of experience (*p* < 0.001). Additionally, mental health strain varied significantly by age group (*p* < 0.001) and experience (*p* < 0.01), while turnover intentions differed significantly across all demographic variables (*p* < 0.001; 0.05), except education level (*p* > 0.05) (Table 1).

**Table 1:** Characteristics of the participants and differences in the study variables.

Characteristics	*N*	%	Professional burnout	Mental health strain	Turnover intentions
			Mean (SD)	Mean (SD)	Mean (SD)
Age					
20–30	139	59.4	3.94 (0.75)	1.65 (0.39)	3.51 (1.11)
31–40	56	23.9	3.77 (0.70)	1.59 (0.46)	3.13 (1.04)
> 40	39	16.7	3.25 (0.80)	1.31 (0.42)	2.42 (1.07)
Mean ± SD	31.28 ± 8.04				
f/p			13.01/< 0.001	10.49/< 0.001	15.69/< 0.001
Gender					
Male	26	11.1	4.06 (0.71)	1.64 (0.40)	3.74 (1.00)
Female	208	88.9	3.75 (0.79)	1.58 (0.43)	3.17 (1.16)
t/p			1.93/0.06	0.69/0.49	2.41/0.02
Marital status					
Unmarried	77	32.9	3.96 (0.82)	1.65 (0.43)	3.49 (1.05)
Married	157	67.1	3.69 (0.74)	1.54 (0.42)	3.11 (1.18)
t/p			2.53/0.01	1.78/0.08	2.46/0.02
Education					
Technical degree	205	87.6	3.76 (0.79)	1.58 (0.44)	3.20 (1.16)
Bachelor degree	29	12.4	3.87 (0.71)	1.57 (0.39)	3.48 (1.05)
t/p			0.66/0.51	0.11/0.92	1.23/0.22
Experience					
1–5	104	44.4	3.96 (0.71)	1.64 (0.37)	3.53 (1.07)
6–10	41	17.5	3.94 (0.82)	1.70 (0.46)	3.55 (1.19)
> 10	89	38.0	3.5 (0.77)	1.46 (0.45)	2.75 (1.07)
Mean ± SD	10.11 ± 8.64				
f/p			10.26/< 0.001	6.68/0.002	14.09/< 0.001

*Note:* t = Independent *t*‐test, f = Analysis of variance.

### 4.2. Descriptive and Correlational Statistics of Study Variables

The mean scores for the primary variables were as follows: professional burnout (*M* = 3.78, SD = 0.78), mental health strain (*M* = 1.58, SD = 0.43), and turnover intentions (*M* = 3.24, SD = 1.15).

Professional burnout exhibited a strong positive correlation with turnover intentions (*r* = 0.585, *p* < 0.001) and a moderate correlation with mental health strain (*r* = 0.446, *p* < 0.001). Moreover, mental health strain correlated significantly with turnover intentions (*r* = 0.503, *p* < 0.001), suggesting interconnectedness among the constructs (Table 2).

**Table 2:** Descriptive statistics and correlation analysis.

The study variables	Mean ± SD	1	2	3
(1) Professional burnout	3.78 ± 0.78	1		
(2) Mental health strain	1.58 ± 0.43	0.446^∗∗∗^	1	
(3) Turnover intentions	3.24 ± 1.15	0.585^∗∗∗^	0.503^∗∗∗^	1

*Note:* ^∗∗∗^ *p* < 0.001.

### 4.3. Mediating Effect of Mental Health Strain on the Linkage of Professional Burnout With Turnover Intentions

**Figure 2:**
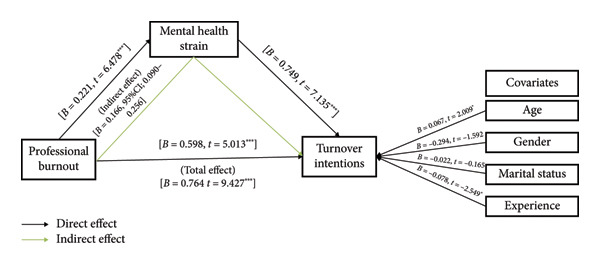
The mediation path of professional burnout on turnover intentions through mental health strain with controlling participants’ demographics as covariates.

Burnout demonstrated a significant direct positive effect on mental health strain (*B* = 0.221, *p* < 0.001) and turnover intentions (*B* = 0.589, *p* < 0.001). Mental health strain was also a significant predictor of turnover intentions (*B* = 0.749, *p* < 0.001). The indirect effect of burnout on turnover intentions through mental health strain was statistically significant (*B* = 0.166; 95% CI [0.090–0.256]), indicating partial mediation. Among covariates, age had a small but significant positive association with turnover intentions (*B* = 0.067, *p* < 0.05), while experience exhibited a modest but significant negative effect (*B* = −0.078, *p* < 0.05) (Table 3 and Figure 2).

**Table 3:** Estimates from the mediation analysis.

Direct effects	*B*	SE	*t*	Bootstrapping 95% CI	
				LLCI	ULCI
Covariates					
Age ⟶ MHS	0.006	0.015	0.377	−0.023	0.035
Gender ⟶ MHS	0.031	0.082	0.373	−0.131	0.192
Marital status ⟶ MHS	−0.008	0.058	0.135	−0.122	0.107
Experience ⟶ MHS	−0.012	0.014	0.870	−0.039	0.015
Age ⟶ TIs	0.067	0.033	2.009^∗^	0.001	0.132
Gender ⟶ TIs	−0.294	0.185	1.592	−0.658	0.069
Marital status ⟶ TIs	−0.022	0.131	0.165	−0.279	0.23
Experience ⟶ TIs	−0.078	0.031	2.549^∗^	−0.139	−0.018
PB ⟶ MHS	0.221	0.034	6.478^∗∗∗^	0.154	0.288
MHS ⟶ TIs	0.749	0.149	7.135^∗∗∗^	0.433	0.764
PB ⟶ TIs	0.598	0.033	5.013^∗∗∗^	0.455	1.044
Indirect effects					
PB ⟶ MHS ⟶ TIs	0.166	0.042		0.090	0.256
Total effect	0.764	0.081	9.427^∗∗∗^	0.605	0.924

*Note:* ^∗^ *p* < 0.05, ^∗∗∗^ *p* < 0.001.

Abbreviations: MHS = Mental health strain, PB = Professional burnout, TIs = Turnover intentions.

## 5. Discussion

This study examined the impact of professional burnout on nurses’ turnover intentions, with a particular focus on the mediating role of mental health strain.

The findings revealed a significant positive association between burnout and nurses’ intentions to leave their jobs, thereby confirming Hypothesis 1. This suggests that higher levels of burnout increase the likelihood of turnover intentions among nurses. This result aligns with prior studies indicating that burnout is a major predictor of nurses’ intent to leave their roles. For instance, a study of Said and El‐Shafei [11] conducted in Zagazig City, Egypt, reported that the highly stressful work environment in triage hospitals led to increased job dissatisfaction and a higher tendency among nurses to consider leaving their positions. Similarly, in Saudi Arabia, Al‐Mansour [49] found that stress was positively associated with turnover intentions among healthcare workers. In Jordan, Oweidat et al. [50] showed that nurses in isolation units faced significant stressors leading to increased turnover intentions. In the Chinese healthcare context, Zhang et al. [51] demonstrated that job stress had a greater direct effect on turnover intention than job satisfaction and organizational commitment.

Supporting Hypothesis 2, burnout was positively associated with mental health strain. This aligns with extensive literature indicating that burnout contributes to psychological distress, including anxiety, depression, and emotional exhaustion [19, 21, 29]. For instance, Gniewek et al. [20] found that burnout adversely affected job satisfaction and quality of care among Polish nurses, highlighting its broader psychological and professional implications.

This study found that mental health strain was significantly associated with turnover intentions. This finding suggests that nurses who experience heightened psychological distress are more likely to consider leaving their positions, supporting H3. The COR theory provides a useful framework to interpret this result. According to the COR theory, prolonged exposure to workplace stress depletes nurses’ emotional and psychological resources, prompting withdrawal as a coping mechanism. This result is in line with previous research indicating that poor mental health is a strong predictor of turnover in healthcare organizations [31]. For instance, Qin et al. [52] found that emotional fatigue, anxiety, and depression significantly increased nurses’ intentions to leave the profession. Similarly, Zhang et al. [51] reported that diminished well‐being reduced retention among Chinese nurses. Other studies have further emphasized the roles of understaffing, job dissatisfaction, and burnout in predicting nurses’ intentions to resign [6].

Supporting Hypothesis 4, mental health strain significantly mediated the relationship between burnout and turnover intentions. This finding indicates that psychological distress serves as a mechanism through which burnout indirectly fosters turnover intentions, emphasizing its role as a proximal outcome in the burnout‐turnover pathway. While the observed effect size was moderate, its practical significance should not be underestimated. Reducing burnout may alleviate psychological strain and consequently lower the risk of attrition. This finding highlights the importance of early identification of psychological distress and the implementation of preventive strategies, such as workload management, resilience training, and access to counseling services to mitigate turnover. Importantly, the mediating role of mental health strain identifies a modifiable mechanism through which healthcare organizations can improve retention.

These findings are congruent with the JD‐R model [15], which posits that excessive job demands, such as emotional exhaustion and depersonalization deplete personal resources, resulting in reduced well‐being and increased turnover intentions. Nurses experiencing burnout often report emotional detachment and diminished professional efficacy, factors that undermine organizational commitment and increase turnover likelihood. This is consistent with studies by Mousavi et al. [53], who identified a strong correlation between burnout dimensions (emotional exhaustion, depersonalization, and low personal accomplishment) and psychological symptoms such as depression and anxiety. Similarly, Andlib et al. [21] reported that burnout significantly contributes to mental distress among Pakistani nurses. Pang et al. [54] and Qin et al. [52] further confirmed that chronic emotional exhaustion is a key driver of nurse turnover.

Existing literature underscores the central role of mental health in mediating the impact of adverse working conditions on nurses’ career decisions. For instance, mental health factors such as stress and anxiety have been shown to mediate the relationship between workplace violence and turnover intentions [55–57]. These findings collectively suggest that sustained exposure to psychosocial stressors within the work environment can erode mental well‐being, ultimately driving decisions to exit the profession.

Furthermore, the mediating role of mental health strain underscores a modifiable psychological mechanism through which healthcare organizations can improve nurse retention. However, the experience and expression of burnout and mental distress are deeply influenced by cultural norms. In collectivist societies such as Egypt, where social conformity, respect for authority, and familial obligations are highly emphasized, nurses may be culturally conditioned to suppress emotional exhaustion and avoid seeking psychological support due to prevailing stigma and fear of reputational harm [58, 59]. These sociocultural dynamics often lead to underreporting of mental health challenges, internalization of stress, and delayed coping, thereby intensifying psychological strain and indirectly increasing turnover intentions. Similar trends have been observed in other Arab and Middle Eastern contexts. For instance, Elshamy et al. [60] found that stigma and cultural beliefs significantly hinder mental health help‐seeking behaviors, exacerbating burnout and increasing turnover intentions among healthcare workers. Also, Said and El‐Shafei [11] found that cultural stigmatization of mental health concerns in Egypt may inhibit nurses from pursuing necessary psychological support, thereby exacerbating burnout and contributing to higher turnover intentions during health crises.

In addition to psychological variables, demographic factors also influence turnover intentions. Older nurses were more likely to express intentions to leave, potentially reflecting proximity to retirement. This aligns with findings from Markowski et al. [61], who reported that age significantly influences older nurses’ career exit decisions. Conversely, greater professional experience was associated with reduced turnover intentions. Experienced nurses may possess better coping strategies, stronger clinical competencies, and deeper organizational commitment, all of which enhance job retention. This finding is consistent with research by Ma et al. [62], who found that longer work experience and greater organizational commitment reduce turnover intentions among emergency nurses. Mirzaei et al. [63] also identified demographic factors such as gender, marital status, and job role as significant predictors of nurses’ turnover intentions. Wu et al. [64] highlighted that younger, single, male nurses with less education and fewer work hours are more susceptible to leaving the profession. These insights highlight the complex interplay between personal and professional variables in shaping nurses’ career trajectories.

## 6. Limitations and Further Research

This study has several limitations that must be acknowledged. First, the use of a cross‐sectional design restricts the ability to infer causal relationships among professional burnout, mental health strain, and turnover intentions. The findings indicate associations rather than causal effects. Longitudinal studies are recommended to more robustly assess temporal and directional dynamics within these constructs. Second, reliance on self‐reported data introduces potential response biases, including social desirability and recall bias. Future research could incorporate multisource data, such as supervisor ratings or administrative turnover records, to triangulate findings and enhance measurement validity.

Third, the sample was drawn exclusively from Egyptian hospitals, which limits the generalizability of the findings to broader international contexts. Healthcare systems vary considerably across countries in terms of staffing norms, leadership practices, work culture, and mental health support infrastructure. Cultural factors such as collectivist orientations, stigma around mental health, and hierarchical organizational structures may influence how nurses experience and report burnout and psychological strain. Consequently, caution is warranted when applying these findings beyond the Egyptian context. Future comparative or cross‐cultural studies are essential to examine whether similar associations exist across diverse health systems and sociocultural environments.

Fourth, while the GHQ‐12 has been widely employed in occupational health research, its original purpose as a screening tool for transient distress may not fully capture the long‐term nature of mental health strain as conceptualized in this study. Future investigations should consider complementary tools that assess chronic psychological strain or persistent emotional depletion over time to enrich construct precision. Fifth, this study focused on testing the mediation effect of mental health strain between burnout and turnover intention, and data collection was limited to basic demographic variables. Detailed information on nurses’ units, professional titles, or income was not collected. Future research should include stratified samples and more detailed professional data to explore variations across departments.

Finally, although mental health strain was investigated as a key mediating mechanism, other important psychological and organizational pathways may exist. Future studies could explore additional mediators such as job satisfaction, organizational commitment, or emotional exhaustion, and examine moderating variables including perceived social support, resilience, ethical leadership, and organizational justice. These factors may be associated with variations in the relationship between burnout and turnover intentions and provide practical levers for organizational intervention.

## 7. Practical Implications

Based on the study’s findings, healthcare institutions should prioritize the enhancement of mental health support programs, the fostering of a supportive work environment, and the implementation of burnout prevention strategies to mitigate the negative effects of burnout and mental health strain on turnover intentions. Key strategies include ensuring adequate staffing levels, offering flexible scheduling options, promoting supportive leadership, and providing access to psychological counseling services [65]. Supportive leadership is essential for mitigating workplace stress and promoting a culture of well‐being among nurses. Nurse managers and administrators should implement leadership strategies that prioritize open communication, emotional support, and professional development opportunities. By empowering nurses and recognizing their contributions, healthcare organizations can enhance feelings of belonging and job satisfaction, potentially decreasing burnout‐related turnover intentions. Furthermore, incorporating mental health resources into organizational policies, such as resilience training and stress management workshops, can provide nurses with effective coping strategies. Emphasizing mental health and well‐being initiatives will not only enhance nurse retention and job satisfaction but also ensure the provision of high‐quality patient care.

## 8. Conclusion

This study provides empirical evidence that nurses experience excessive levels of burnout, moderate mental health strain, and moderate to high turnover intentions. Professional burnout turned into discovered to noticeably impact both mental health strain and turnover intentions among nurses. Furthermore, mental strain acts as a mediator within the relationship between professional burnout and turnover intentions, highlighting the critical want for centered interventions aimed at helping nurses’ mental well‐being. These findings underscore the significance of addressing burnout and its mental outcomes to enhance retention and promote a healthy work environment for nursing profession.

Beyond organizational‐level interventions, comprehensive policy actions are imperative to address the structural determinants of nurse burnout and mental health challenges. Health policymakers should prioritize the implementation of evidence‐based national standards for nurse‐to‐patient staffing ratios, institutionalize access to mental health support services, and allocate sustained funding for workplace wellness programs. Furthermore, integrating routine mental health assessments into occupational health protocols and enacting legislation that safeguards and promotes nurses’ psychological well‐being are essential steps toward mitigating burnout. These systemic strategies are critical to fostering a sustainable, psychologically resilient nursing workforce capable of meeting the evolving demands of healthcare systems.

## Ethics Statement

Ethical clearance was granted for the study by Research Ethics Committee at Damietta University (Reference Number: DU Rec no 55; dated: 29/12/2024). In accordance with the principles of the Declaration of Helsinki, informed consent was obtained from all participants prior to their involvement in the study.

## Disclosure

All authors have approved the final version for submission.

## Conflicts of Interest

The authors declare no conflicts of interest.

## Author Contributions

All authors have met at least one of the authorship criteria outlined by the latest guidelines of the International Committee of Medical Journal Editors (ICMJE). Each author has made substantial contributions to the conception, design, data collection, analysis, or interpretation of the study and has participated in drafting or critically revising the manuscript. Furthermore, all authors agree to be accountable for all aspects of the work.

## Funding

This work was supported and funded by the Deanship of Scientific Research at Imam Mohammad Ibn Saud Islamic University (IMSIU) (grant number IMSIU‐DDRSP2501).

## Supporting Information

This file includes copies of the study instruments used for data collection.

## Supporting information


**Supporting Information** Additional supporting information can be found online in the Supporting Information section.

## Data Availability

The data that support the findings of this study are available upon request from the corresponding author. The data are not publicly available due to privacy or ethical restrictions.

## References

[1] WHO , Nursing and Midwifery: Key Facts, 2022, https://www.who.int/news-room/fact-sheets/detail/nursing-and-midwifery.

[2] Jun J. , Ojemeni M. M. , Kalamani R. , Tong J. , and Crecelius M. L. , Relationship Between Nurse Burnout, Patient and Organizational Outcomes: Systematic Review, International Journal of Nursing Studies. (2021) 119, 10.1016/j.ijnurstu.2021.103933. 33901940

[3] Majrabi M. , Nurses Burnout, Resilience and Its Association With Safety Culture: A Cross Sectional Study, Open Journal of Nursing. (2022) 12, no. 1, 70–102, 10.4236/ojn.2022.121006.

[4] Li L. Z. , Yang P. , Singer S. J. , Pfeffer J. , Mathur M. B. , and Shanafelt T. , Nurse Burnout and Patient Safety, Satisfaction, and Quality of Care: A Systematic Review and Meta-Analysis, JAMA Network Open. (2024) 7, no. 11, 10.1001/jamanetworkopen.2024.43059. PMC11539016 39499515

[5] Muir K. J. , Wanchek T. N. , Lobo J. M. , and Keim-Malpass J. , Evaluating the Costs of Nurse Burnout-Attributed Turnover: A Markov Modeling Approach, Journal of Patient Safety. (2022) 18, no. 4, 351–357, 10.1097/PTS.0000000000000920. 35617593

[6] Haywood D. , Crocker K. M. , Gnatt I. et al., What Accounts for Turnover Intention in the Australian Public Mental Health Workforce?, International Journal of Mental Health Nursing. (2024) 33, no. 2, 359–368, 10.1111/inm.13233. 37795874

[7] Bae S. , Noneconomic and Economic Impacts of Nurse Turnover in Hospitals: A Systematic Review, International Nursing Review. (2022) 69, no. 3, 392–404, 10.1111/inr.12769. 35654041 PMC9545246

[8] Anwar M. M. and Elareed H. R. , Burnout Among Egyptian Nurses, Journal of Public Health. (2017) 25, no. 6, 693–697, 10.1007/s10389-017-0831-2, 2-s2.0-85034770238.

[9] Ibraheim O. G. , Shehata A. I. , and Elhoseny T. A. , Burnout and Associated Risk Factors Among Nurses Working in COVID-19 Isolation Hospitals: A Cross-Sectional Study in Egypt, Journal of the Egyptian Public Health Association. (2025) 100, no. 1, 10.1186/s42506-025-00192-0. PMC12069197 40353982

[10] Hamed R. A. , Abd Elaziz S. Y. , and Ahmed A. S. , Prevalence and Predictors of Burnout Syndrome, Post-Traumatic Stress Disorder, Depression, and Anxiety in Nursing Staff in Various Departments, Middle East Current Psychiatry. (2020) 27, no. 1, 10.1186/s43045-020-00044-x.

[11] Said R. M. and El-Shafei D. A. , Occupational Stress, Job Satisfaction, and Intent to Leave: Nurses Working on Front Lines During COVID-19 Pandemic in Zagazig City, Egypt, Environmental Science and Pollution Research. (2021) 28, no. 7, 8791–8801, 10.1007/s11356-020-11235-8. 33067794 PMC7567651

[12] Abdo S. A. M. , El-Sallamy R. M. , El-Sherbiny A. A. M. , and Kabbash I. A. , Burnout Among Physicians and Nursing Staff Working in the Emergency Hospital of Tanta University, Egypt, Eastern Mediterranean Health Journal. (2015) 21, no. 12, 906–915, 10.26719/2015.21.12.906, 2-s2.0-84961590345. 26996364

[13] Mosallam R. , Hamidi S. , and Elrefaay M. , Turnover Intention Among Intensive Care Unit Nurses in Alexandria, Egypt, Journal of the Egyptian Public Health Association. (2015) 90, no. 2, 46–51, 10.1097/01.EPX.0000464696.41556.EB, 2-s2.0-84942517530. 26154830

[14] Abbas A. , Ali A. , Bahgat S. , and Shouman W. , Prevalence, Associated Factors, and Consequences of Burnout Among ICU Healthcare Workers: An Egyptian Experience, Egyptian Journal of Chest Diseases and Tuberculosis. (2019) 68, no. 4, 10.4103/ejcdt.ejcdt_188_18.

[15] Demerouti E. , Bakker A. B. , Nachreiner F. , and Schaufeli W. B. , The Job Demands-Resources Model of Burnout, Journal of Applied Psychology. (2001) 86, no. 3, 499–512, 10.1037/0021-9010.86.3.499, 2-s2.0-85047685234. 11419809

[16] Hobfoll S. E. , Conservation of Resources: A New Attempt at Conceptualizing Stress, American Psychologist. (1989) 44, no. 3, 513–524, 10.1037/0003-066X.44.3.513, 2-s2.0-0024632747. 2648906

[17] Kristensen T. S. , Borritz M. , Villadsen E. , and Christensen K. B. , The Copenhagen Burnout Inventory: A New Tool for the Assessment of Burnout, Work & Stress. (2005) 19, no. 3, 192–207, 10.1080/02678370500297720, 2-s2.0-31944434614.

[18] Dall’Ora C. , Ball J. , Reinius M. , Griffiths P. , and Griffiths P. , Burnout in Nursing: A Theoretical Review, Human Resources for Health. (2020) 18, 1–18, 10.1186/s12960-020-00469-9. 32503559 PMC7273381

[19] Wang L. , Zhang X. , Zhang M. et al., Risk and Prediction of Job Burnout in Responding Nurses to Public Health Emergencies, BMC Nursing. (2024) 23, no. 1, 10.1186/s12912-024-01714-5. PMC10792923 38233880

[20] Gniewek D. , Wawro W. , Czapla M. , Milecka D. , Kowalczuk K. , and Uchmanowicz I. , Occupational Burnout Among Nursing Professionals: A Comparative Analysis of 1103 Polish Female Nurses Across Different Hospital Settings, Sustainability. (2023) 15, no. 11, 10.3390/su15118628.

[21] Andlib S. , Inayat S. , Azhar K. , and Aziz F. , Burnout and Psychological Distress Among Pakistani Nurses Providing Care to COVID-19 Patients: A Cross-Sectional Study, International Nursing Review. (2022) 69, no. 4, 529–537, 10.1111/inr.12750. 35167710 PMC9111774

[22] Sharma P. R. and Sharma R. , Burnout in Oncology Nurses, Journal of Clinical Oncology. (2023) 41, no. 16_suppl, 10.1200/jco.2023.41.16_suppl.e23000.

[23] Tolksdorf K. H. , Tischler U. , and Heinrichs K. , Correlates of Turnover Intention Among Nursing Staff in the COVID-19 Pandemic: A Systematic Review, BMC Nursing. (2022) 21, no. 1, 10.1186/s12912-022-00949-4. PMC9252069 35787700

[24] Cheung A. T. , Ho L. L. K. , Li W. H. C. , Chung J. O. K. , and Smith G. D. , Psychological Distress Experienced by Nurses Amid the Fifth Wave of the COVID-19 Pandemic in Hong Kong: A Qualitative Study, Frontiers in Public Health. (2023) 10, 10.3389/fpubh.2022.1023302. PMC9880411 36711417

[25] Bruyneel A. , Bouckaert N. , de Noordhout C. M. et al., Association of Burnout and Intention-to-Leave the Profession With Work Environment: A Nationwide Cross-Sectional Study Among Belgian Intensive Care Nurses After Two Years of Pandemic, International Journal of Nursing Studies. (2023) 137, 10.1016/j.ijnurstu.2022.104385. PMC9640385 36423423

[26] Zhang J. , Lu J. , Zhao S. et al., Developing the Psychological Strain Scales (PSS): Reliability, Validity, and Preliminary Hypothesis Tests, Social Indicators Research. (2014) 115, no. 1, 337–361, 10.1007/s11205-012-0222-6, 2-s2.0-84892480368. 24443628 PMC3891678

[27] Dobson M. and Schnall P. L. , From Stress to Distress: The Impact of Work on Mental Health, Unhealthy Work: Causes, Consequences, Cures. (2018) Routledge, Abingdon, 113–132, 10.4324/9781315223421-9/STRESS-DISTRESS-IMPACT-WORK-MENTAL-HEALTH-MAMIE-DOBSON-PETER-SCHNALL.

[28] Sung-Man B. , The Influence of Strain due to Individual Risk Factors and Social Risk Factors on Depressive Symptoms and Suicidality-A Population-Based Study in Korean Adults: A STROBE-Compliant Article, Medicine. (2018) 97, no. 27, 10.1097/MD.0000000000011358, 2-s2.0-85049984311. PMC6076168 29979418

[29] Chen C. and Meier S. T. , Burnout and Depression in Nurses: A Systematic Review and Meta-Analysis, International Journal of Nursing Studies. (2021) 124, 10.1016/j.ijnurstu.2021.104099. 34715576

[30] Tabakakis C. , Bradshaw J. , McAllister M. , and Sahay A. , Psychological Distress in Registered Nurses and the Role of the Workplace: A Cross-Sectional Study, Australian Journal of Advanced Nursing. (2024) 41, no. 3, 10.37464/2024.413.980.

[31] Foster K. , Shakespeare‐Finch J. , Shochet I. et al., Psychological Distress, Well‐Being, Resilience, Posttraumatic Growth, and Turnover Intention of Mental Health Nurses During COVID‐19: A Cross‐Sectional Study, International Journal of Mental Health Nursing. (2024) 33, no. 5, 1543–1552, 10.1111/inm.13354. 38747675

[32] Edwin H. S. , Trinkoff A. M. , Mills M. E. , and Zhu S. , Psychological Distress Symptoms in Nurses and Their Intention to Leave: A Cross-Sectional Secondary Data Analysis, Journal of Advanced Nursing. (2025) 81, no. 3, 1286–1299, 10.1111/JAN.16290. 39031572

[33] Anees R. T. , Heidler P. , Cavaliere L. P. L. , and Nordin N. A. , Brain Drain in Higher Education. The Impact of Job Stress and Workload on Turnover Intention and the Mediating Role of Job Satisfaction at Universities, European Journal of Business and Management Research. (2021) 6, no. 3, 1–8, 10.24018/ejbmr.2021.6.3.849.

[34] Lapointe É. , Vandenberghe C. , and Panaccio A. , Organizational Commitment, Organization-Based Self-Esteem, Emotional Exhaustion and Turnover: A Conservation of Resources Perspective, Human Relations. (2011) 64, no. 12, 1609–1631, 10.1177/0018726711424229, 2-s2.0-82355184695.

[35] Korbus H. , Hildebrand C. , Schott N. et al., Health Status, Resources, and Job Demands in Geriatric Nursing Staff: A Cross-Sectional Study on Determinants and Relationships, International Journal of Nursing Studies. (2023) 145, 10.1016/j.ijnurstu.2023.104523. 37327686

[36] Bakker A. B. and Demerouti E. , The Job Demands‐Resources Model: State of the Art, Journal of Managerial Psychology. (2007) 22, no. 3, 309–328, 10.1108/02683940710733115, 2-s2.0-33947307639.

[37] Obeng A. F. , Quansah P. E. , and Boakye E. , The Relationship Between Job Insecurity and Turnover Intention: The Mediating Role of Employee Morale and Psychological Strain, Management Science. (2021) 10, 35–45.

[38] Giorgi G. , Perminiene M. , Montani F. , Fiz-Perez J. , Mucci N. , and Arcangeli G. , Detrimental Effects of Workplace Bullying: Impediment of Self-Management Competence via Psychological Distress, Frontiers in Psychology. (2016) 7, 1–11, 10.3389/fpsyg.2016.00060, 2-s2.0-84978152165. 26913013 PMC4753400

[39] Williams P. and Goldberg D. P. , A User’s Guide to the General Health Questionnaire, 1988, NFER.

[40] Gómez-Salgado J. , Domínguez-Salas S. , Romero-Martín M. , Romero A. , Coronado-Vázquez V. , and Ruiz-Frutos C. , Work Engagement and Psychological Distress of Health Professionals During the COVID-19 Pandemic, Journal of Nursing Management. (2021) 29, no. 5, 1016–1025, 10.1111/JONM.13239;PAGE:STRING:ARTICLE/CHAPTER. 33400325

[41] Ren Z. , Zhao H. , Zhang X. et al., Associations of Job Satisfaction and Burnout With Psychological Distress Among Chinese Nurses, Current Psychology. (2022) 42, no. 33, 10.1007/S12144-022-04006-W. PMC9662123 36406845

[42] Eweida R. S. , Rashwan Z. I. , Desoky G. M. , and Khonji L. M. , Mental Strain and Changes in Psychological Health Hub Among Intern-Nursing Students at Pediatric and Medical-Surgical Units amid Ambience of COVID-19 Pandemic: A Comprehensive Survey, Nurse Education in Practice. (2020) 49, 10.1016/J.NEPR.2020.102915. PMC7655025 33227694

[43] Moosa F. , Chontawan R. , and Akkadechanunt T. , Factors Related to Intent to Stay Among Nurses in the Tertiary Hospital, Maldives, Nursing Journal. (2016) 43, 129–142.

[44] Brislin R. W. , Back-Translation for Cross-Cultural Research, Journal of Cross-Cultural Psychology. (1970) 1, no. 3, 185–216, 10.1177/135910457000100301, 2-s2.0-84965932242.

[45] Nunnally J. C. and Bernstein I. H. , Psychometric Theory, 1994, 3rd edition, McGraw-Hill.

[46] Hu L. and Bentler P. M. , Cutoff Criteria for Fit Indexes in Covariance Structure Analysis: Conventional Criteria Versus New Alternatives, Structural Equation Modeling. (1999) 6, 1–55, 10.1080/10705519909540118, 2-s2.0-67650706330.

[47] Field A. , Discovering Statistics Using IBM SPSS Statistics, 2024, Sage Publications Limited.

[48] Kim H.-Y. , Statistical Notes for Clinical Researchers: Assessing Normal Distribution (2) Using Skewness and Kurtosis, Restorative Dentistry & Endodontics. (2013) 38, no. 1, 10.5395/rde.2013.38.1.52. PMC3591587 23495371

[49] ‏ and Al-Mansour K. , Stress and Turnover Intention Among Healthcare Workers in Saudi Arabia During the Time of COVID-19: Can Social Support Play a Role?, PLoS One. (2021) 16, no. 10, 10.1371/journal.pone.0258101. PMC8496805 34618851

[50] Oweidat I. A. , Abu Shosha G. M. , Omoush O. A. et al., Work Stressors and Intention to Leave Among Nurses in Isolation Nursing Units During COVID-19: A Cross-Sectional Study, BMC Nursing. (2025) 24, no. 1, 10.1186/s12912-025-02779-6. PMC11827187 39948518

[51] Zhang D. , Qin L. , Huang A. et al., Mediating Effect of Resilience and Fear of COVID-19 on the Relationship Between Social Support and Post-Traumatic Stress Disorder Among Campus-Quarantined Nursing Students: A Cross-Sectional Study, BMC Nursing. (2023) 22, 1–9, 10.1186/s12912-023-01319-4. 37193966 PMC10185929

[52] Qin N. , Yao Z. , and Guo M. , The Role of Bidirectional Associations Between Depression, Anxiety, and Emotional Exhaustion on Turnover Intention Among Nurses: A Multicenter Cross-Sectional Study in China, BMC Nursing. (2023) 22, no. 1, 10.1186/s12912-023-01516-1. PMC10548568 37789287

[53] Mousavi S. V. , Ramezani M. , Salehi I. , Hossein Khanzadeh A. A. , and Sheikholeslami F. , The Relationship Between Burnout Dimensions and Psychological Symptoms (Depression, Anxiety and Stress) Among Nurses, Journal of Holistic Nursing and Midwifery. (2017) 27, no. 2, 37–43, 10.18869/acadpub.hnmj.27.2.37.

[54] Pang Y. , Dan H. , Jung H. , Bae N. , and Kim O. , Depressive Symptoms, Professional Quality of Life and Turnover Intention in Korean Nurses, International Nursing Review. (2020) 67, no. 3, 387–394, 10.1111/inr.12600. 32633425

[55] Adiguna A. A. B. W. and Suwandana I. G. M. , The Relationship Between Burnout, Work Stress, and Turnover Intention on Non-Permanent (Contract) Employees: Study at the Communication and Information Office of Badung Regency, Indonesia, European Journal of Business and Management Research. (2023) 8, no. 3, 104–107, 10.24018/ejbmr.2023.8.3.1964.

[56] Salama W. , Abdou A. H. , Mohamed S. A. K. , and Shehata H. S. , Impact of Work Stress and Job Burnout on Turnover Intentions Among Hotel Employees, International Journal of Environmental Research and Public Health. (2022) 19, no. 15, 10.3390/IJERPH19159724. PMC9368148 35955081

[57] Chen Y. , Wang P. , Zhao L. et al., Workplace Violence and Turnover Intention Among Psychiatrists in a National Sample in China: The Mediating Effects of Mental Health, Frontiers in Psychiatry. (2022) 13, 10.3389/FPSYT.2022.855584/BIBTEX. PMC9240432 35782425

[58] Huwaë S. and Schaafsma J. , Cross-Cultural Differences in Emotion Suppression in Everyday Interactions, International Journal of Psychology. (2018) 53, no. 3, 176–183, 10.1002/IJOP.12283, 2-s2.0-84969980462. 27168184

[59] Al-amer R. M. , Al weldat K. , Ali A. et al., Arab Nursing Students’ Perception of the Emotional Experience of Patient Care: A Phenomenological Study, Nursing Forum. (2022) 57, no. 6, 1176–1183, 10.1111/NUF.12818. 36315113

[60] Elshamy F. , Hamadeh A. , Billings J. , and Alyafei A. , Mental Illness and Help-Seeking Behaviours Among Middle Eastern Cultures: A Systematic Review and Meta-Synthesis of Qualitative Data, PLoS One. (2023) 18, no. 10, 10.1371/JOURNAL.PONE.0293525. PMC10602270 37883515

[61] Markowski M. , Cleaver K. , and Weldon S. M. , An Integrative Review of the Factors Influencing Older Nurses’ Timing of Retirement, Journal of Advanced Nursing. (2020) 76, no. 9, 2266–2285, 10.1111/jan.14442. 32500926

[62] Ma Y. , Chen F. , Xing D. , Meng Q. , and Zhang Y. , Study on the Associated Factors of Turnover Intention Among Emergency Nurses in China and the Relationship Between Major Factors, International Emergency Nursing. (2022) 60, 10.1016/J.IENJ.2021.101106. 34864323

[63] Mirzaei A. , Rezakhani Moghaddam H. , and Habibi Soola A. , Identifying the Predictors of Turnover Intention Based on Psychosocial Factors of Nurses During the COVID-19 Outbreak, Nursing Open. (2021) 8, no. 6, 3469–3476, 10.1002/nop2.896. 33960721 PMC8242757

[64] Wu F. , Lao Y. , Feng Y. , Zhu J. , Zhang Y. , and Li L. , Worldwide Prevalence and Associated Factors of Nursing Staff Turnover: A Systematic Review and Meta-Analysis, Nursing Open. (2024) 11, 1–10, 10.1002/nop2.2097. PMC10802134 38268271

[65] Norful A. A. , Cato K. , Chang B. P. , Amberson T. , and Castner J. , Emergency Nursing Workforce, Burnout, and Job Turnover in the United States: A National Sample Survey Analysis, Journal of Emergency Nursing. (2023) 49, no. 4, 574–585, 10.1016/J.JEN.2022.12.014. 36754732 PMC10329980

