# Supplementary material for: Exploring the Role of Professional Burnout and Mental Health Strain in Nurses’ Turnover Intentions: A Mediation Study
Source: J Nurs Manag. 2025 Dec 16;2025:1175290. doi: 10.1155/jonm/1175290 (PMC12714161; doi:10.1155/jonm/1175290)
Supplement: Supplementary file 1 — Supporting Information Additional supporting information can be found online in the Supporting Information section. [file JONM-2025-1175290-s001.docx]

**أولا: مقياس الإرهاق المهني**

1. هل تشعر بالإرهاق في نهاية يوم العمل؟

2. هل تشعر بالإرهاق في الصباح عند التفكير في يوم عمل جديد؟

3. هل تشعر أن كل ساعة عمل مرهقة بالنسبة لك؟

4. هل تملك طاقة كافية لقضائها مع العائلة والأصدقاء خلال أوقات الفراغ؟ )معكوس الترميز)

5. هل عملك مرهق عاطفيًا؟

6. هل عملك يسبب لك الإحباط؟

7. هل تشعر بالإرهاق بسبب عملك؟

**ثانيا : مقياس الصحة النفسية العامة**

1. هل تستطيع التركيز فيما تقوم به؟

2. هل فقدت الكثير من النوم بسبب القلق؟

3. هل تشعر أنك تقوم بدور مفيد في الحياة؟

4. هل تشعر بأنك قادر على اتخاذ القرارات؟

5. 5. هل تشعر أنك تحت ضغط مستمر؟

6. هل تشعر بأنك غير قادر على التغلب على صعوباتك؟

7. هل تستطيع الاستمتاع بأنشطتك اليومية المعتادة؟

8. هل تستطيع مواجهة مشاكلك؟

9. هل تشعر بالحزن والاكتئاب؟

10. هل بدأت تفقد الثقة بنفسك؟

11. هل بدأت ترى نفسك كشخص عديم القيمة؟

12. هل شعرت بأنك سعيد إلى حد معقول، بالنظر إلى جميع الأمور؟

**ملاحظة: العناصر 1، 3، 4، 7،6، 8(معكوسة الترميز)**

**ثالثا : مقياس نوايا ترك العمل**

1. أتوقع أن أغادر هذه المستشفى خلال السنة القادمة.

2. أرغب في العمل في مكان آخر غير هذه المستشفى.

3. أرغب في مغادرة هذه المستشفى.

**رابعا : الصفات الشخصية:**

**العمر:**

**النوع :**

1. ذكر ( )
2. أنثي ( )

**الحالة الاجتماعية:**

1. غير متزوج ( )
2. متزوج ( )

**المستوي التعليمي:**

1. معهد تمريض ( )
2. بكالوريوس تمريض ( )
3. دراسات عليا ( )

**عدد سنوات الخبرة:**

**Frist: Work-related Burnout**

1. Do you feel exhausted at the end of the workday?
2. Are you exhausted in the morning at the thought of another day at work?
3. Do you feel that every working hour is tiring for you?
4. Do you have enough energy for family and friends during leisure time? (reverse coded)
5. Is your work emotionally exhausting?
6. Does your work cause you frustration?
7. Do you feel burnt out because of your work?

**Second: General Health Questionnaire**

1. Are you able to concentrate on what you are doing? (reverse coded)
2. Have you lost much sleep due to worry?
3. Do you feel that you are playing a useful role in life? (reverse coded)
4. Do you feel capable of making decisions? (reverse coded)
5. Do you feel under constant pressure?
6. Do you feel unable to overcome your difficulties? (reverse coded)
7. Are you able to enjoy your daily activities? (reverse coded)
8. Are you able to face your problems? (reverse coded)
9. Do you feel sad and depressed?
10. Have you begun to lose confidence in yourself?
11. Have you begun to see yourself as a worthless person?
12. Have you felt reasonably happy, considering all things?

**Third: Turnover Intention Scale**

1. I expect to leave this hospital within the next year.
2. I would like to work somewhere other than this hospital.
3. I would like to leave this hospital.

**Fourth: Demographic Characteristics**

**Age:**

**Gender:**

1. Male ( )
2. Female ( )

**Marital Status:**

1. Unmarried ( )
2. Married ( )

**Educational Level:**

1. Nursing institute ( )
2. Bachelor of Nursing ( )
3. Postgraduate / Higher Studies ( )

**Years of Experience:** _____
